# Urea-Based Patches with Controlled Release for Potential Atopic Dermatitis Treatment

**DOI:** 10.3390/pharmaceutics14071494

**Published:** 2022-07-19

**Authors:** Zuzanna J. Krysiak, Urszula Stachewicz

**Affiliations:** Faculty of Metals Engineering and Industrial Computer Science, AGH University of Science and Technology, 30-059 Krakow, Poland; krysiak@agh.edu.pl

**Keywords:** PVB, electrospinning, electrospray, fibers, urea

## Abstract

Skin diseases such as atopic dermatitis (AD) are widespread and affect people all over the world. Current treatments for dry and itchy skin are mostly focused on pharmaceutical solutions, while supportive therapies such as ointments bring immediate relief. Electrospun membranes are commonly used as a drug delivery system, as they have a high surface to volume area, resulting in high loading capacity. Within this study we present the manufacturing strategies of skin patches using polymer membranes with active substances for treating various skin problems. Here, we manufactured the skin patches using electrospun poly(vinyl butyral-co-vinyl alcohol-co-vinyl acetate) (PVB) fibers blended and electrosprayed with urea. The highest cumulative release of urea was obtained from the PVB patches manufactured via blend electrospinning with 5% of the urea incorporated in the fiber. The maximum concentration of released urea was acquired after 30 min, which was followed up by 6 h of constant release level. The simultaneous electrospinning and electrospraying limited the urea deposition and resulted in the lowest urea incorporation followed by the low release level. The urea-based patches, manufactured via blend electrospinning, exhibited a great potential as overnight treatment for various skin problems and their development can bring new trends to the textile-based therapies for AD.

## 1. Introduction

Skin barrier disfunction, affecting people regardless of age, leads to transepidermal water loss and allergens penetration, which accelerate the course of a disease such as atopic dermatitis (AD) [1]. The symptoms are dry, itchy skin with a bacterial infection, which is difficult to treat and usually requires using different products for each symptom [2]. Non-pharmaceutical treatments minimize pharmaceutical ones and their frequent side effects. The most common are bathing techniques, ointments, and moisturizers, but also textiles-based therapies, such as silver-impregnated textiles, cotton clothing [3], and overnight wet wraps [4]. Topical treatments require reapplication during the day; therefore, materials with prolonged controlled active agent release are desirable [5]. Electrospun fibers were applied as a patch for skin hydration improvement [6]. They were loaded with evening primrose, borage or blackcurrant seed oil and used as reservoirs, which slowly released the oil and moisturized the skin [7,8,9]. Moreover, electrospun fibers are widely used as drug delivery system with controlled release [10]. Electrospinning enables the production of fibers from various polymers and with different geometries [11] and tunable surface properties [12,13] that can be controlled via electrospinning parameters [14,15] that strictly influence active molecule release [16]. Core-shell [17], side-by-side [18] or blend electrospinning can be applied to obtain drug-loaded electrospun membranes [19]. For instance, ampicillin was blended with polycaprolactone (PCL) and loaded, as a core of fiber, while the shell was PCL. The antibiotic release was compared between core-shell fibers and those made from core solution. After 4 h, 84% of ampicillin was released from PCL and the antibiotic blend, while only 16% was released from the core-shell material. This demonstrates that the PCL shell layer retarded the ampicillin release and presented a sustained drug release manner [20]. Post-processing of electrospun fibers also gives an opportunity for drug incorporation into the electrospun membrane. Cellulose fibers were immersed in the ibuprofen solution and then dried. The total drug release took 5 h, due to a lack of chemical interaction between the polymer and medicine [21]. Furthermore, the degradation rate of the polymer and type of its solvent also have an impact on drug delivery system properties. In constructing drug release patches, we need to take into consideration the selection of the solvent regarding the polymer and drug properties. Polymers soluble in water have a short degradation time, which is followed by burst drug release [22]. Urea is broadly used in dermatology, as it improves the skin barrier and acts as a moisturizer and keratolytic agent [23]. It is highly water-soluble, and incorporated in 4–10% formulations to moisturize skin; thus it is often applied in AD treatment [24]. Two different formulations, containing 5% and 10% of urea, were tested in subjects with AD. They were applied twice a day for six weeks and could improve mild to moderate AD. Interestingly, both products were well tolerated and showed a similar effect on the skin [25]. Furthermore, non-detergent urea and detergent cleansers were used to increase skin hydration and reduce transepidermal water loss (TEWL). Both types of detergents demonstrated comparable results; however, skin improvement caused by an urea-based formulation lasted significantly longer [26].

Within this study, we selected poly(vinyl butyral-co-vinyl alcohol-co-vinyl acetate) (PVB) nanofibers, as they showed great skin hydration in vivo, while loaded with the evening primrose oil [7]. Thus, PVB fibrous membrane can be applied as a multifunctional dressing with additional modification using other skin curing substances. Here, we tested methods of urea incorporation into the PVB fibers by blending and electrospraying. The produced patches were characterized in terms of morphology, chemical composition, thermal and wetting properties. We verified the biocompatibility of urea-based patches with keratinocytes and performed the release tests to select the most effective strategies for applying urea into patches for easy and comfortable use for patients not only with AD.

## 2. Materials and Methods

### 2.1. Preparation of the Urea-Based Patches

The urea-based patches were prepared using two methods; the first one was to blend electrospinning of the PVB and the urea and the second was simultaneous electrospinning of PVB fibers and electrospraying of the urea.

### 2.2. Blend Electrospinning of PVB and Urea

The 2 wt% and 5 wt% solution of urea was prepared in the mixture of methanol (MeOH), *N*,*N*-dimethyloformamide (DMF) and dimethyl sulfoxide (DMSO); solvents were analytical standard (Avantor, Poland), mixed in a ratio of 5:4:1 and stirred at 500 rpm on a magnetic stirrer plate (IKA RCT basic, Staufen, Germany) for 15 min in 25 °C. Then, poly(vinyl butyral-co-vinyl alcohol-co-vinyl acetate) (PVB, 70,000–100,000 g·mol^−1^, Sigma-Aldrich, St. Louis, MO, USA) was added to both solutions up to 10 wt% and stirred for 3 h at 1000 rpm in 35 °C. Fibers were produced via electrospinning (IME Technologies, The Netherlands) in a climate control set at T = 25 °C and RH = 30%. To produce nanofibers, the high voltage of 16–17 kV was applied to the horizontally moving stainless steel needle (20 mm·s^−1^) with an inner diameter of 0.5 mm and outer one of 0.8 mm. The distance between the needle and slowly rotating drum (5 rpm) was kept at 15 cm. The polymer flow rate was 1.0 mL·h^−1^ and fibers were collected for 1 h on the baking paper.

### 2.3. Simultaneous Electrospinning of PVB and Electrospraying of Urea

10 wt% urea and 10 wt% PVB solution were prepared, in MeOH and MeOH, DMF and DMSO mixed in a ratio of 5:4:1, respectively. The electrospinning parameters were the same, as described above. However, two needles were used with a solution’s flow rate of 1.0 mL·h^−1^ for the PVB and 0.45 mL·h^−1^ for the urea.

For the patches’ production purpose, the conductivity of solutions: pristine PVB, 2% and 5% urea and PVB blend and 10% urea in methanol were measured using Mettler Toledo Conductometer (SevenCompact S210, Zurich, Switzerland) equipped with a conductivity probe (InLab 720). The measurements were performed in triplicate, at T = 21 °C and RH = 30%.

### 2.4. Characterization of Urea-Based Patches

#### 2.4.1. Membrane Morphology

The morphology of all the manufactured samples was analyzed with a scanning electron microscope (SEM, Phenom ProX—Desktop Scanning Electron Microscopy, Thermo Fisher Scientific, Waltham, MA, USA) with accelerated voltage of 10 kV using a backscattered electron. Before imaging, the samples were sputtered with 8 nm gold layer (Q150RS, Quorum Technologies, Lewes, UK). Fiber diameters were measured from the SEM micrographs using ImageJ software (ver.1.53v, National Institutes of Health, Bethesda, MD, USA) and the average fiber diameter within the standard error values was calculated from 100 measurements [27].

#### 2.4.2. Wetting

The water contact angle tests were performed with deionized water (DI water, Spring 5UV purification system Hydrolab, Straszyn, Poland) on studied membranes. Images were taken with a Canon EOS 700D camera with an EF-S 60 mm f/2.8 Macro USM zoom lens immediately after pipetting 3 μL of water droplets on the membrane. The contact angle was analyzed using a MB-Ruler (ver. 5.3, Iffezheim, Germany) and the mean value was calculated from 10 measurements; errors are based on standard deviation [11].

#### 2.4.3. Fourier-Transform-Infrared Spectroscopy (FTIR) and Differential Scanning Calorimetry (DSC)

Spectra of pristine PVB nanofibers blended with 2% and 5% urea and electrosprayed with urea were recorded on a Nicolet iS5 FT-IR spectrophotometer (Thermo Fisher Scientific, Waltham, MA, USA), by the ATR technique using the diamond crystal. During measurements, the spectra were 64 repeated over the wavenumber range 500–4000 cm^−1^, with a resolution of 4 cm^−1^ [28,29]. As reference samples, PVB fibers and pristine urea were used.

Thermal characterization was carried out using a DSC (Mettler Toledo, DSC 3, Columbus, OH, USA) at a heating rate of 10 °C·min^−1^ in the 20 to 250 °C temperature range. Measurements were carried out in a dynamic N_2_ atmosphere for the sample placed in Al pans [30,31].

### 2.5. In Vitro Urea Release

Urea release from electrospun fibers was determined by placing samples (5 × 5 cm) into the test tube filled with 2 mL of deionized water and incubated in a shaker (25 °C, 200 rpm, Shaker IKA KS 3000 IC Control, Staufen, Germany). The supernatant was collected after 5, 15, 30, 60, 120, 240, 360, 480 min. In each test, 1 mL of the sample was taken and replaced with fresh deionized water. The released urea was detected with Urea Nitrogen (BUN) Colorimetric Detection Kit (Invitrogen, Thermo Fisher Scientific, Waltham, MA, USA), and the concentration was calculated from the standard curve [32].

### 2.6. Biocompatibility

Prior to the assay, PVB nanofibers both blended and electrosprayed with the urea, were placed in the 24-well plate and sterilized with UV light. All sample types were incubated for 5, 15, 30, 60, 120, 240, 360, 480 min at 37 °C, RH = 90% and 5% of CO_2_ in 1 mL complete cell culture medium composed of Dulbecco’s modified Eagle medium (DMEM with 4.5 g/L D- glucose, Biological Industries, Kibbutz Beit-Haemek, Israel), supplemented with 10% of fetal bovine serum (FBS, Biological Industries, Israel), 2% of antibiotics (penicillin−streptomycin, Biological Industries, Kibbutz Beit-Haemek, Israel), 1% of L-glutamine solution (Biological Industries, Kibbutz Beit-Haemek, Israel), and 1% of aminoacids (Mem nonessential amino acid solution 100×, Sigma-Aldrich, St. Louis, MO, USA). After each time point, the medium was transferred to the test tube and stored at 4 °C until use. Human immortalized (HaCaT cell line) [33] were seeded on the 24-well plate with the density of 5 × 10^4^ cells per well and cultured for 24 h. Then, the medium was removed, and cells were incubated with collected supernatants for 24 h; for each solution three repetitions were performed. After that time, 600 µL of supernatant was discarded and 80 μL of CellTiter Blue (Promega, Madison, WI, USA) reagent was added to each well to verify the cell proliferation. It was incubated for 4 h at 37 °C, H = 90% and 5% CO_2_ (Memmert GmbH + Co.KG, INC 108med, Schwabach, Germany). Next, 100 μL of each reaction solution was transferred to the 96-well plate in four repetitions, and the fluorescence was measured (excitation 560 nm/emission 590 nm) (GloMax Discover, Promega, Madison, WI, USA). Analysis of variance (one-way ANOVA followed by Tukey’s post-hoc test) was used to determine the level of significance between the urea-based patches and the Tissue Culture Polystyrene (TCPS) for cell proliferation; the statistical significance was evaluated at *p* < 0.05 [34].

## 3. Results and Discussion

### 3.1. Fiber Morphology

**Figure 1:**
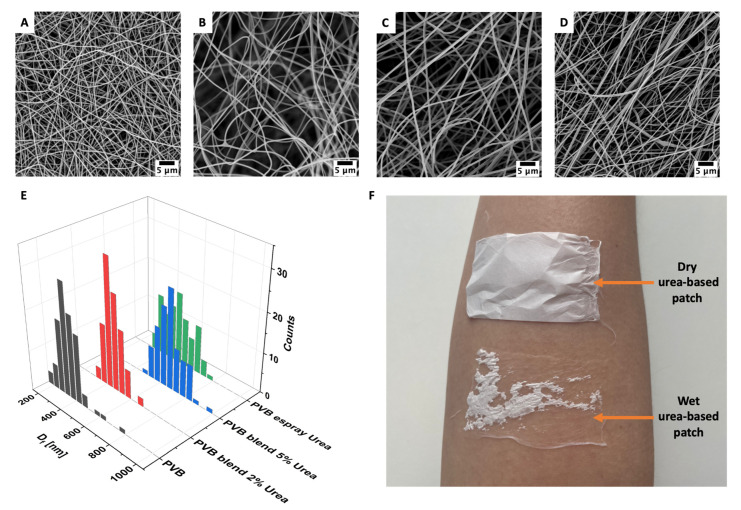
SEM micrographs of electrospun nanofibers, (**A**) PVB, (**B**) PVB blend with 2% of urea, (**C**) PVB blend with 5% of urea, (**D**) PVB fibers electrosprayed with urea solution. (**E**) The fiber diameter distribution showed in histogram. (**F**) Images of patches made of PVB blend with 5% urea, dry and sprayed with water, placed on the skin of the forearm.

The morphology of PVB fibers was analyzed with SEM as shown in Figure 1, and the average diameter was 347 ± 87 nm, similar to the previous study [34]. The incorporation of the urea into the two types of patches produced, via blend electrospinning and electrospraying increased the average fiber diameter to 455 ± 67 nm and 672 ± 96 nm, respectively for samples with 2% and 5% urea in the PVB blend, and 518 ± 112 nm for PVB membranes with the electrosprayed solution containing 10% of urea (see Figure 1E). During electrospinning, water from the air could bind to the urea and became entrapped in the fibers, resulting in a larger diameter of manufactured PVB fibers [35].

In terms of spinnability of urea-based patches, the conductivity of polymer solutions was verified. The conductivity of the PVB solution after adding the urea slightly increased from 7.5 ± 0.1 μS·cm^−1^ for pristine PVB to 12.1 ± 0.1 μS·cm^−1^ and 15.7 ± 0.1 μS·cm^−1^, respectively for 2% and 5% of urea in the polymer blend. The conductivity of the 10% urea solution in methanol, which was used for electrospraying, reached 12.9 ± 0.1 μS·cm^−1^.

Next the wettability was verified; the contact angle for the PVB membrane was 130.7° ± 2.1°; for the blended PVB with 2% (Figure 1B) and 5% urea (Figure 1C) it settled at, 128.1° ± 1.6° and 122.4° ± 2.4°, respectively, and for the PVB fibers electrosprayed with the urea solution (Figure 1D) it was 126.3° ± 1.9°. As the urea is a water-soluble molecule, its addition may change the surface chemistry of electrospun membranes, having an effect on its wetting too. All PVB patches with the urea kept their hydrophobic properties; however, when the patches were applied to the skin and sprayed with water, the membranes were firmly attached to the skin. Water was immediately soaked and spread between the fibers. However, the dry urea-based patches stuck out from the skin as presented in Figure 1F, indicating the applicability of electrospun urea-based dressings.

Within this study, we selected 10% urea solution as it was reported previously to have the greatest effect in improving skin hydration [36]. However, the electrospinning of the PVB blend with 10% urea was unstable without reproducible results. Thus, we reduced the concentration of the urea to 5% and 2% in PVB blends, which resulted in a stable electrospinning process allowing us the collection of reproducible samples. On the contrary, for the electrospraying the solution of 10% urea in methanol was characterized by a stable deposition; therefore, this maximum concentration of urea was used for the production of the patches. Due to the low urea deposition efficiency via electrospraying, the lower concentration of urea (5% and 2%) in the solution was not considered in further studies.

### 3.2. Chemical Analysis—FTIR and DSC

**Figure 2:**
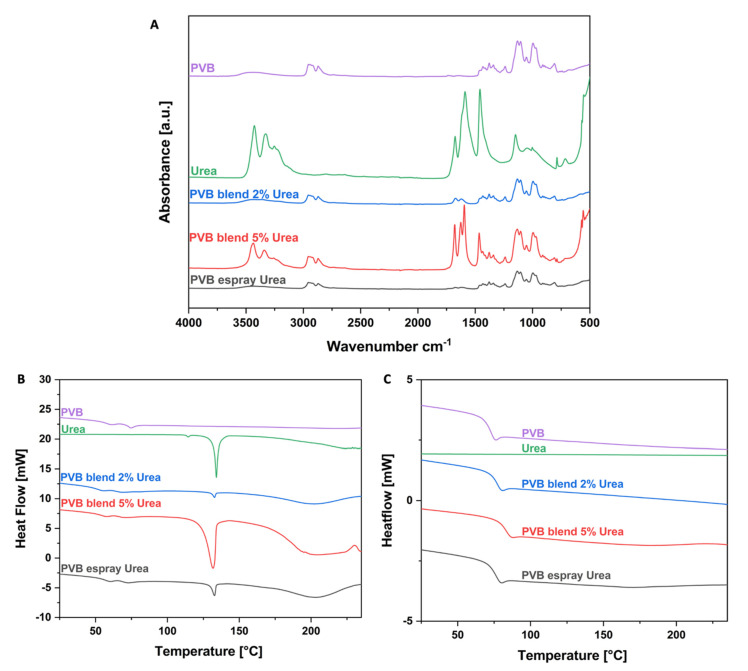
(**A**) The ATR-FTIR spectra of: urea powder, pristine PVB fibers, PVB blend with 2% and 5% of urea and PVB fibers electrosprayed with urea solution. DSC thermograms, (**B**) the first and (**C**) the second heating scan of PVB and urea powder, pristine PVB fibers, PVB blend with 2% and 5% of urea and PVB fibers electrosprayed with urea solution.

Chemical analysis of electrospun patches was performed to confirm the urea presence in the fibers and verify interaction between the polymer and the urea. The FTIR spectra of PVB and urea powder, pristine PVB fibers, blended and electrosprayed with urea, are presented in Figure 2A. The analysis of the urea showed that the stretching frequency of C=O appeared at 1678 cm^−1^ and for C–N at 1459 cm^−1^. The N–H bond was detected at 3427 cm^−1^ (stretching) and at 1589 cm^−1^ (deformation) [28]. For PVB, at 3100–3600 cm^−1^ there was a flat peak corresponding to the –OH group. The stretching frequency of C–H appeared at 2750–3000 cm^−1^, while bending at 1300–1500 cm^−1^. Typical bonds of PVB were presented at 1132 cm^−1^ and 999 cm^−1^, respectively corresponding to the butyral ring (C–O–C) and C–O stretching [29,37]. FTIR spectra analysis confirms urea presence in all PVB patches. In the 5% urea and PVB blend, the intensity of peaks related to the urea is the highest, while compared to the 2% blend and electrosprayed membrane. The bond C=O was found at 1679 cm^−1^ and C–N at 1466 cm^−1^. Stretching and deformation frequency of the N–H bond appeared at 3440 cm^−1^ and 1587 cm^−1^, respectively, thus indicating that no peaks shifted due to the polymer and urea blending. Moreover, the same peaks were detected for the 2% blend and membranes electrosprayed with the urea solution, also without shifting. These peaks had lower intensity, while compared to the 5% blend. The characteristic peaks for the PVB were detected without shifting. Therefore, the C–H bond was observed at 2750–3000 cm^−1^ (stretching) and 1300–1500 cm^−1^ (bending). Urea addition to the polymer led to H–bond formation between the urea and the ether or hydroxyl group, resulting in a reduction in C–H peak intensity [35]. The representative frequencies of PVB remain at the same wavelengths as for pristine PVB fibers, C–O–C at 1132 cm^−1^ and C–O at 999 cm^−1^. The stretching frequency of N–H in the urea at around 3400 cm^−1^ and the bond related to the –OH group in the PVB were difficult to observe, as they were in the similar range. This result indicates that the chemical composition of both the urea and the PVB was not affected by the processing conditions used to manufacture patches.

In Figure 2B,C, we present the DSC curves for the first and second heating cycle for the urea powder, pristine PVB fibers and those blended and electrosprayed with urea. The difference between the first and second cycle of samples heating is noticeable, as two peaks for the PVB samples near the glass transition temperature (Tg) are indicated in Figure 2B, but in Figure 2C only one peak is presented. Electrospinning used for membrane production affecting the polymer chain alignment in fibers resulted in the structural changes of the produced materials that are represented by the glass transition temperature variation [38,39]. For PVB fibers, Tg reached 74 °C, while for membranes containing urea it was slightly reduced. The peaks for the 2%, 5% blend and electrosprayed with urea solution were at 71 °C, 69 °C and 72 °C, respectively [40,41]. The peak at 132 °C corresponds to the melting temperature of the urea (see Figure 2B). Above this temperature, urea decomposition occurred [42]; thus, this peak disappeared in the second heating cycle (see Figure 2C). The DSC results confirmed the FTIR spectra; the 5% urea in the PVB blend has the highest peak intensity, hence the amount of urea was the greatest in this type of patch.

### 3.3. In Vitro Urea Release and Biocompatibility

**Figure 3:**
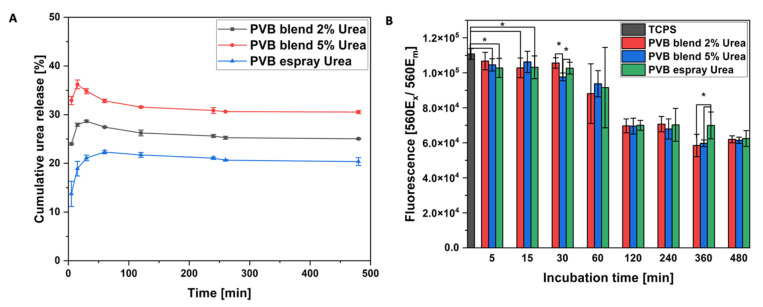
(**A**) Cumulative urea release from PVB fibers blended and electrosprayed with urea, (**B**) Biocompatibility test after 24 h of keratinocytes cultured with the medium incubated with PVB fibers blended and electrosprayed with urea for 5, 15, 30, 60, 120, 240, 360, 480 min; TCPS was used as a positive control. * Statistical significance calculated with ANOVA, followed by Tukey’s post hoc test, *p* < 0.05; error bars are based on standard deviation. All the samples incubated for 30 min and more showed statistically significant difference with TCPS.

The cumulative urea release from PVB fibers was investigated for up to 6 h (see Figure 3A). After 30 min, the concentration of released urea reached its maximum and was then followed by a plateau. Urea was added to the mixture of PVB solvents: MeOH, DMF and DMSO. However, among these three solvents, it was soluble only in MeOH; therefore, phase separation during electrospinning occurred and most of the urea was deposited near or on the fibers’ surface [43], resulting in the fast release. The greatest release, about 35% of the urea, was obtained for the patches based on the PVB blend with 5% of the urea, which had the highest initial urea content (see Figure 3). The total urea release did not occur, as the urea is bound to the polymer through the H– bond [35], occurring in electrospun blend PVB patches, as also indicated by FTIR results (see Figure 2A).

The indirect biocompatibility test was performed with keratinocytes. Up to 30 min, the urea released to the cell culture medium did not significantly reduce HaCaT proliferation, while compared to the TCPS (see Figure 3B). Within the increasing urea concentration, the cell number decreased, reaching less than half of the positive control. After 60 min, the amount of released urea showed cytotoxic effects for cells and reduced their proliferation [44]. After 2 h, the number of cells incubated with the supernatant from electrosprayed patches reached a stable level. The electrosprayed urea on PVB fibers was easily released, as it was placed on their surface, while the urea blended in PVB fibers was captured inside fibers too. Finally, the cell proliferation was reduced for all the samples on a similar level, as there was only a slight difference in the urea cumulative release between the samples (Figure 3A). This phenomenon can be considered as a drawback for tissue engineering; however, the direct biocompatibility of PVB has already been verified with fibroblasts (NIH 3T3) and keratinocytes (HaCaT) [7,34]. Thus, in this study, the decreased cell proliferation is strictly correlated with the urea presence, as the number of keratinocytes started to be reduced, when the urea reached the maximum concentration (Figure 3A). Here, we electrospun patches for topical application, aiming to have contact with the epidermis, where the biocompatible of polymer itself is most important. Urea is widely used in various skin applications, especially in topical wound treatment due to its antibacterial properties [45,46]. It also helps with various dermatoses causing dry and scaly skin [24,36]. Previously, the urea was blended with poly(vinyl alcohol) and papain (proteolytic enzyme) to electrospun patches for wound debridement, showing antibacterial properties against E.coli—the most commonly found in the acute and chronic lesions of wounds [32]. Considering other applications, the controlled release of the urea for agriculture purposes was obtained from wheat gluten electrospun fibers immersed in the urea solution [30]. Both examples showed the possibility of urea incorporation into electrospun membranes via a different method. In our studies, we compared two approaches that can be used for incorporating the urea in electrospun patches. One is blend electrospinning and the other is a combination of electrospinning and electrospraying. Summarizing, we observed the greatest urea release from the PVB blend with the 5% urea as the electrospraying even a higher concentration (10%) of urea in the solution is limited by the small droplets being incorporated in the membrane. The initial amount of incorporated urea restricts the released portion. The initial burst release was related to the urea, which did not form the bond with the polymer and was deposited on the surface of PVB fibers. After 2 h, all the investigated urea-based patches reached a stable release level (Figure 3A).

## 4. Conclusions

Electrospun fibers are an excellent material for active molecule delivery, especially in the form of skin patches. In this study, PVB fibers were successfully loaded with urea by blending and electrospraying. The highest cumulative release of the urea was obtained from the patches manufactured via blend electrospinning with 5% of the urea incorporated in the PVB fibers. The simultaneous electrospinning and electrospraying limited the urea deposition and resulted in the lowest urea incorporation followed by a low release level. PVB fibers have excellent biocompatibility; however, here the cytotoxic effect was induced by the cells’ direct contact with the urea. Although the proliferation of keratinocytes was reduced, the viability tests confirmed the suitability of the urea-based patches for topical application. Importantly, the PVB fibers blended with the urea, reaching the maximum urea release after 30 min, giving the immediate effect on skin treatment after wetting the patch on the skin. After that the release level of the urea is constant during a 6 h treatment. It indicates great suitability of the urea-based patches to be used as an overnight dressing without reapplication needs. The preparation strategies of skin patches presented here indicate the enormous potential of combining polymer membranes with active substances for treating various skin problems.

## Data Availability

The data supporting this article are found within the text.
